# ZnT8-Specific CD4^+^ T Cells Display Distinct Cytokine Expression Profiles between Type 1 Diabetes Patients and Healthy Adults

**DOI:** 10.1371/journal.pone.0055595

**Published:** 2013-02-04

**Authors:** Daisuke Chujo, Emile Foucat, Thien-Son Nguyen, Damien Chaussabel, Jacques Banchereau, Hideki Ueno

**Affiliations:** 1 Baylor Institute for Immunology Research, Dallas, Texas, United States of America; 2 Benaroya Research Institute at Virginia Mason, Seattle, Washington, United States of America; La Jolla Institute for Allergy and Immunology, United States of America

## Abstract

Determination of antigen-specific T cell repertoires in human blood has been a challenge. Here, we show a novel integrated approach that permits determination of multiple parameters of antigen-specific T cell repertoires. The approach consists of two assays: the Direct assay and the Cytokine-driven assay. Briefly, human PBMCs are first stimulated with overlapping peptides encoding a given antigen for 48 hours to measure cytokine secretion (Direct assay). Peptide-reactive T cells are further expanded by IL-2 for 5 days; and after overnight starvation, expanded cells are stimulated with the same peptides from the initial culture to analyze cytokine secretion (Cytokine-driven assay). We first applied this integrated approach to determine the type of islet-antigen-specific T cells in healthy adults. Out of ten donors, the Direct assay identified GAD65-specific CD4^+^ T cells in three adults and zinc transporter 8 (ZnT8)-specific CD4^+^ T cells in five adults. The intracytoplasmic cytokine staining assay showed that these islet-antigen-specific CD4^+^ T cells belonged to the CD45RO^+^ memory compartment. The Cytokine-driven assay further revealed that islet-antigen-specific CD4^+^ T cells in healthy adults were capable of secreting various types of cytokines including type 1 and type 2 cytokines as well as IL-10. We next applied our integrated assay to determine whether the type of ZnT8-specific CD4^+^ T cells is different between Type 1 diabetes patients and age/gender/HLA-matched healthy adults. We found that ZnT8-specific CD4^+^ T cells were skewed towards Th1 cells in T1D patients, while Th2 and IL-10-producing cells were prevalent in healthy adults. In conclusion, the Direct assay and the Cytokine-driven assay complement each other, and the combination of the two assays provides information of antigen-specific T cell repertoires on the breadth, type, and avidity. This strategy is applicable to determine the differences in the quality of antigen-specific T cells between health and disease.

## Introduction

The increased incidence of immune-mediated diseases and the growing numbers of immunomodulatory agents that are moving from bench to bedside demand development of better tools to study the human immune system. In particular, identifying the type of antigen-specific T cell responses may provide significant insights regarding disease pathogenesis and lead to the development of novel platforms for therapeutic approaches. Yet, the enormous diversity of immune responses renders the assessment of the antigen-specific T cell repertoires a challenge. This is particularly true in humans due to the extensive HLA polymorphism. Currently, several tools are available to identify antigen-specific T cells, including the proliferation assay [1], [2], ELISPOT [1], [3], [4], intracytoplasmic cytokine staining (ICS) [5], [6], [7], and peptide-HLA tetramers [1], [4], [8], [9], [10]. Although all assays permit the detection of antigen-specific T cells in human blood, each assay displays both strengths and shortcomings. [^3^H]-thymidine incorporation or the CFSE dilution assay can be performed at a low-cost [1], [2], but provide little functional information. ELISPOT assays are widely used to quantitate low-frequency antigen-specific T cells [1], [3], [4], but only one or two soluble factors can be measured in a single assay. ICS assays, in particular when performed with multicolor flow cytometry, permit the detailed assessment of cytokine expression profiles of antigen-specific T cells together with cell phonotype, [5], [6], [7]; though the sensitivity is limited. Peptide-HLA tetramers sensitively determine the frequency of antigen-specific T cells in blood [1], [4], [8], [9], [10], but provide little information regarding their functions.

Overlapping peptides that span a given antigen’s sequence are widely used in multiple platforms to stimulate antigen-specific T cells in vitro irrespective of the HLA alleles. We have previously shown that measurement of multiple cytokines in PBMC cultures stimulated for 48 h with overlapping peptides permits the detection of antigen-specific T cells (here called the Direct assay) [11], [12]. In studies with blood samples from melanoma patients, tumor-antigen-specific IFN-γ-secreting CD8^+^ T cells were identified through the secretion of IP-10 [11], a chemokine that is induced in response to IFN-γ. Furthermore, tumor-antigen-specific regulatory T cells were identified through peptide-induced IL-10 secretion [12]. While these studies show that the Direct assay can identify antigen-specific T cells together with some of their functions, the assay will not allow detection of very rare antigen-specific T cells. One approach to circumvent this shortcoming is to expand peptide-reactive CD4^+^ T cells by adding cytokines such as IL-2 to the cultures prior to the assessment of cytokine secretion profiles (here called the Cytokine-driven assay). Using this strategy, we were able to demonstrate the presence of islet-antigen-specific CD4^+^ and CD8^+^ T cells in Type 1 diabetes (T1D) patients under immunosuppressive treatments after allogeneic islet transplantation [13]. Notably, the Cytokine-driven assay can be combined with the Direct assay in the same cultures, and the combination of the two assays therefore might permit us to characterize multiple aspects of antigen-specific T cell repertoires.

Type 1 diabetes (T1D) is caused by autoimmune destruction of insulin-producing islet β cells [14], [15], [16], [17], [18]. Accordingly, T1D patients display IFN-γ-producing islet antigen-specific T cells in the blood [19], [20], [21]. T1D-associated islet antigens include GAD65 [8], [22], [23], (prepro)insulin (PPI) [24], [25], [26], insulinoma associated-2 (IA-2) [27], islet-specific glucose-6-phosphate catalytic subunit-related protein (IGRP) [28], [29], and zinc transporter-8 (ZnT8) [30]. Previous studies showed that islet-antigen-specific T cells can be also found in healthy adults [20], [31], [32]. Distinct from T1D patients, however, islet-antigen-specific T cells in healthy adults were shown to be either naïve [31], [32] or non-inflammatory cells secreting primarily IL-10 [19].

In this present study, we used our integrated assay to characterize islet-antigen-specific T cell repertoires in healthy adults and in T1D patients. Here, we show that our integrated strategy allowed the detection of GAD65- and ZnT8-specific CD4^+^ T cells in healthy adults as well as in T1D patients. Importantly, the quality of ZnT8-specific CD4^+^ T cells were different between T1D patients and healthy adults. While ZnT8-specific CD4^+^ T cells were skewed towards Th1 cells in T1D patients, Th2 and IL-10-producing cells were prevalent in healthy adults. This study provides a proof-of-principle that our integrated strategy permits determination of multiple parameters of antigen-specific T cell repertoires.

## Results

### The Direct Assay can Detect T cells Recognizing Peptides Derived from Islet-antigens in Healthy Adults

**Figure 1:**
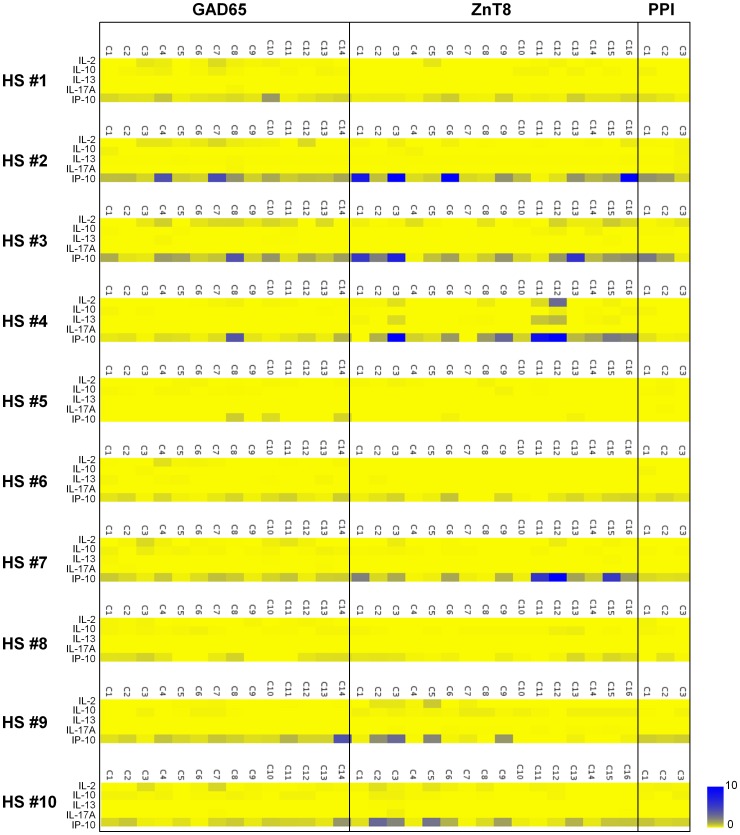
Cytokine secretion profiling of islet-antigen-specific T cells by the Direct assay in healthy adults. PBMCs obtained from ten healthy adults (HS#1–10) were cultured for 48 h with GAD65, ZnT8, and PPI 15-mer peptide clusters (GAD65 C1-C14, ZnT8 C1-C16, and PPI C1-C3). Amounts of IL-2, IL-10, IL-13, IL-17A, and IP-10 in the supernatants were measured by a multiplex bead-based cytokine assay. Cytokine data were transformed into heat-map format indicating the fold increase from the background. Blue indicates the strongest cytokine secretion, whereas yellow indicates the background.

Previous studies showed that healthy individuals also display islet-antigen-specific T cells in the blood [20], [31], [32]. We examined whether our Direct assay is able to identify antigen-specific T cells in healthy adult blood samples. Fresh blood samples were obtained from 10 healthy adults (HS#1–10; 5 male and 5 female, age: 44.8±3.3). Fifteen-mer overlapping peptides were generated for three islet antigens – GAD65, ZnT8, and PPI (Table 1). PBMCs were cultured with peptide pools (4–11 peptides/pool), and cytokines secreted during the 48 h culture were measured. Background levels of IL-2, IL-10, IL-13, and IL-17A in the culture where PBMCs were stimulated with no peptide were less than 10 pg/ml (IL-2∶0.1±0.3, IL-10∶0.8±1.3, IL-13∶0.3±0.5, IL-17A: 0.2±0.4 pg/ml, mean ± SD, *n* = 20). IP-10, a chemokine secreted in response to IFN-γ, was detected at variable levels (159.4±152.3 pg/ml). We defined cytokine secretion in response to stimulation with peptides as “positive”, when 1) the levels of IL-2, IL-10, IL-13, or IL-17A were greater than 10 pg/ml, or 2) the level of IP-10 was more than three times the background. The summary of cytokine levels detected in each culture is shown in Figure 1. The secretion of IL-2, IL-10, IL-13, or IL-17A in response to stimulation with islet-antigen-derived peptides was generally weak, and no cultures scored positive for secretion of IL-10 and IL-17A (range, IL-2 0–103.3 pg/ml, IL-10 0–8.7 pg/ml, IL-13 0–30.1 pg/ml, IL-17A 0–3.46 pg/ml). However, multiple cultures with GAD65 and ZnT8 peptide pools in 5 out of 10 healthy individuals scored positive for IP-10 secretion. PPI-specific T cell responses were not detected in any subjects.

**Table 1:** Numbers of peptides and peptide clusters.

Islet antigen	Number of peptides	Number of peptide clusters	Peptides/cluster
PPI	25	3	8 (C1–C2)
			9 (C3)
GAD65	144	14	10 (C1–C10)
			11 (C11–C14)
ZnT8	94	16	6 (C1–C15)
			4 (C16)

PPI, preproinsulin; GAD65, glutamic acid decarboxylase 65; ZnT8, zinc transporter 8; C, peptide cluster.

**Figure 2:**
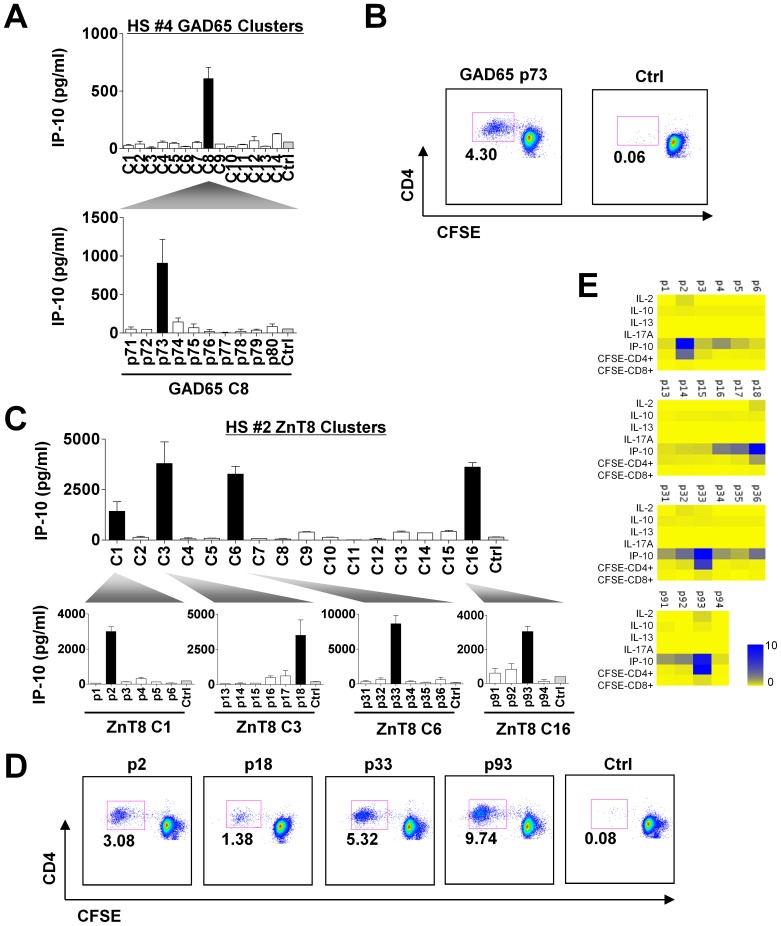
Identification of the epitopes in the Direct assay. CFSE-labeled PBMCs were cultured with single peptides from a “positive” peptide cluster for 8 days. Secreted cytokines were measured on day 2. CD4^+^/CD8^+^ T cell proliferation was assessed on day 8 based on the CFSE dilution. (**A**) Identification of GAD65-specific T cell epitope in HS#4. GAD65 p#73 from peptide cluster #8 (C#8) was found to induce IP-10 secretion. (**B**) Assessment of CD4^+^ T cell proliferation in response to GAD65 p#73 in HS#4. (**C**) Identification of ZnT8-specific T cell epitopes in HS#2. ZnT8 p#2, p#18, p#33, and p#93 were found to induce IP-10 secretion. (**D**) Assessment of CD4^+^ T cell proliferation in response to ZnT8 p#2, p#18, p#33, and p#93 in HS#2. (**E**) Cytokine secretion and T cell proliferation data in the experiments shown in (D) are shown in a heat-map format.

After we identified positive responses at a peptide cluster level, we next cultured PBMCs with single peptides (25 µM) within the positive clusters to identify the peptides that triggered IP-10 secretion. Figure 2A shows an example of results in healthy donor HS#4. GAD65 peptide #73 (p#73) from peptide cluster #8 (C#8) was found to induce IP-10 secretion (Fig. 2A). This peptide was able to induce the proliferation of CD4^+^ T cells in the PBMC culture (Fig. 2B). These results suggest the presence of GAD65-specific Th1 cells in the blood of donor HS#4. In donor HS#2, ZnT8 peptides p#2, p#18, p#33, and p#93 were found to induce IP-10 secretion (Fig. 2C and 2E) as well as CD4^+^ T cell proliferation (Fig. 2D and 2E). Of note, in donor HS#2, while GAD65 peptide #66 was found to induce IP-10 secretion within C#7, no peptides were found within GAD65 peptide C#4. This was likely due to the fact that multiple peptides in C#4 contributed to the secretion of IP-10, but no single peptide was sufficient to score positive for IP-10 secretion. The summary of the identified peptides is shown in Table 2 (The sequence is shown in Table S1). Out of the 10 healthy adults that were tested, 3 adults displayed both GAD65-specific CD4^+^ T cells and ZnT8-specific CD4^+^ T cells. Two adults displayed only ZnT8-specific CD4^+^ T cells. All of the identified peptides induced IP-10 secretion and proliferation of CD4^+^ T cells, suggesting the presence of specific Th1 cells.

**Table 2:** Summary of the islet-antigen-specific T cell responses in the Direct assay.

Subject	Antigen	Cluster	Epitope	Secreted Cytokine					CFSE-CD4^+^	CFSE-CD4^+^
				IL-2	IL-10	IL-13	IL-17	IP-10	(%)	Background (%)
HS#1	–	–	–	–	–	–	–	–	–	–
HS#2	GAD65	C7	p66	–	–	–	–	^+^	5.63	0.26
	ZnT8	C1	p2	–	–	–	–	^+^	3.08	0.11
		C3	p18	–	–	–	–	^+^	1.38	0.11
		C6	p33	–	–	–	–	^+^	5.32	0.11
		C16	p93	–	–	–	–	^+^	9.74	0.11
HS#3	GAD65	C8	p73	–	–	–	–	^+^	1.91	0.03
	ZnT8	C1	p2	–	–	–	–	^+^	2.87	0.03
		C3	p18	–	–	–	–	^+^	1.94	0.03
HS#4	GAD65	C8	p73	^+^	–	–	–	^+^	4.30	0.07
	ZnT8	C3	p18	^+^	–	^+^	–	^+^	9.44	0.07
		C11	p65	^+^	–	–	–	^+^	17.2	0.08
		C12	p68	^+^	–	^+^	–	^+^	47.7	0.08
HS#5	–	–	–	–	–	–	–	–	–	–
HS#6	–	–	–	–	–	–	–	–	–	–
HS#7	ZnT8	C11	p65	–	–	–	–	^+^	1.20	0.21
		C12	p68	^+^	–	–	–	^+^	1.04	0.01
		C15	p87	–	–	–	–	^+^	4.14	0.21
HS#8	–	–	–	–	–	–	–	–	–	–
HS#9	–	–	–	–	–	–	–	–	–	–
HS#10	ZnT8	C2	p8	^+^	–	–	–	^+^	2.62	0.03

HS, healthy subject; C, peptide cluster; p, peptide.

### ICS Assay Confirms the Presence of Th1 Cells Reactive to Islet Antigen-peptides

**Figure 3:**
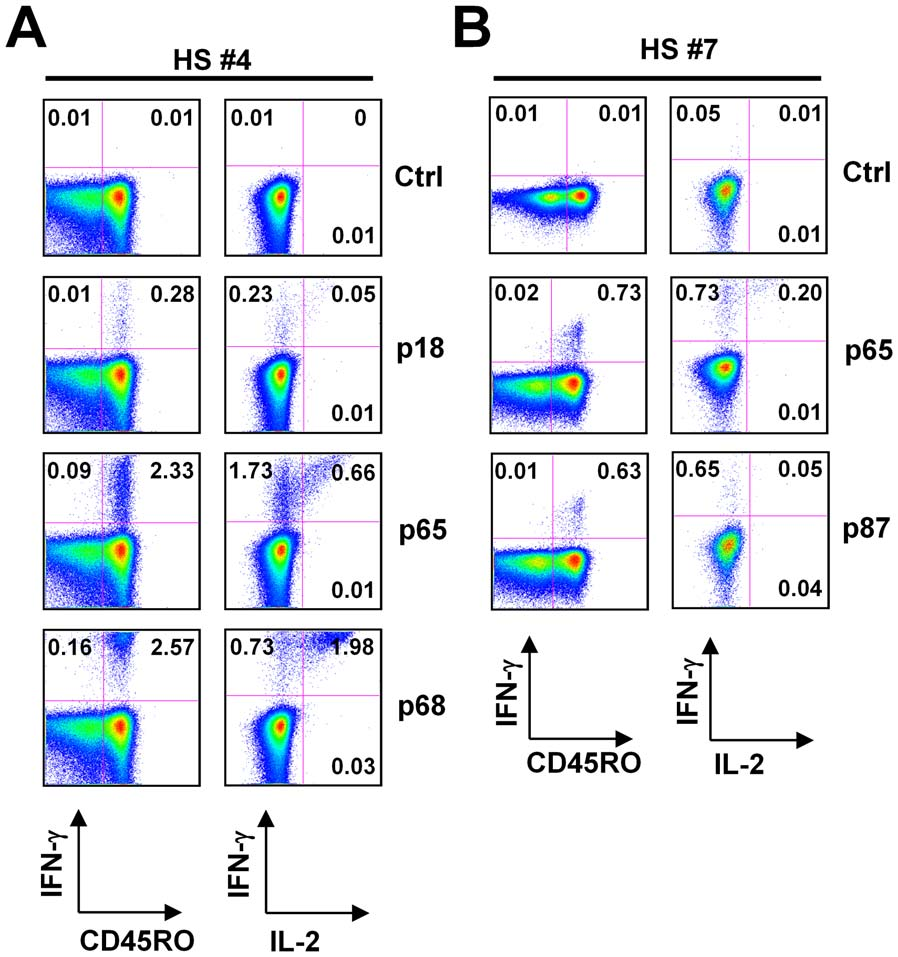
Detection of ZnT8-specific Th1 cells by intracytoplasmic cytokine expression assay. PBMCs were stimulated with the identified peptides in the presence of anti-CD28/CD49d antibody and brefeldin A. After 6 h stimulation, the expression of intracytoplasmic cytokines was analyzed by flow cytometry. Gated to CD3^+^CD4^+^ cells. (**A**) Expression of IFN-γ and IL-2 in CD4^+^ T cells (right) and IFN-γ in CD4^+^CD45RO^+^ memory T cells (left) specific for ZnT8 peptides in HS#4. (**B**) Expression of IFN-γ and IL-2 in CD4^+^ T cells (right) and IFN-γ in CD4^+^CD45RO^+^ memory T cells (left) specific for ZnT8 epitopes in HS#7.

While a recent study showed ZnT8-specific IFN-γ-producing CD4^+^ T cells were present in healthy adults at a lower frequency than in type 1 diabetes patients [21], we were intrigued by the fact that ZnT8 peptides induced strong IP-10 secretion in 5 out of 10 healthy donors (Table 2). To determine the frequency of ZnT8-specific CD4^+^ T cells in healthy adults, PBMCs were incubated with the identified peptide for 6 hours in the presence of Brefeldin A, and intracytoplasmic expression of IFN-γ and IL-2 was analyzed. In donor HS#4, three ZnT8 peptides, p#18, p#65, and p#68 induced IP-10 secretion (Table 2). The ICS assay showed that as many as ∼2.6% of CD4^+^ T cells was found to express IFN-γ in response to p#68 (Fig. 3A). Among IFN-γ-expressing CD4^+^ T cells, a majority also co-expressed IL-2 (Fig. 3A). ∼2.3% and ∼0.3% of CD4^+^ T cells expressed IFN-γ in response to p#65 and p#18, respectively. A quarter of IFN-γ-expressing CD4^+^ T cells cultured with p#65 and p#18 co-expressed IL-2. In donor HS#7, ∼0.7% and ∼0.6% of CD4^+^ T cells expressed IFN-γ in response to ZnT8 p#65 and p#87, respectively (Fig. 3B). While IL-2 was expressed by a fraction of p#65-responding CD4^+^ T cells, few p#87-responding CD4^+^ T cells expressed IL-2. In both donors, IFN-γ was expressed solely by CD45RO^+^ memory T cells (Fig. 3A and 3B).

Thus, ZnT8-specific memory CD4^+^ T cells expressing IFN-γ and IL-2 can be found in the blood of healthy adults at high frequencies (>0.5% of total CD4^+^ T cells). Importantly, this intracytoplasmic cytokine expression assay confirmed the results from the Direct assay.

### The Cytokine-driven Assay Permits Detailed Cytokine Secretion Profiling of Peptide-reactive CD4^+^ T cells

We next used the Cytokine-driven assay to analyze islet-antigen-specific T cells in healthy adults. Briefly, IL-2 was added to the cultures of PBMCs with 25 µM of islet-antigen peptides on day 2. Cells were harvested on day 7, rested overnight in fresh media, and re-stimulated for 24 h with the same peptides. Secreted cytokines were measured with a multiplex cytokine assay. Cytokine secretion in response to stimulation with peptides was defined as “positive”, when the levels of each cytokine were more than ten times those in the background (background values; IL-4 3.9±3.0 pg/ml, IL-5 8.9±6.3 pg/ml, IL-10 10.8±16.5 pg/ml, IL-13 51.2±52.0 pg/ml, IL-17A 3.6±6.6 pg/ml, IL-21 1.9±2.3 pg/ml, TNF-α 45.3±33.2 pg/ml, and IFN-γ: 190.1±280.3 pg/ml; mean ± SD, n = 20).

**Figure 4:**
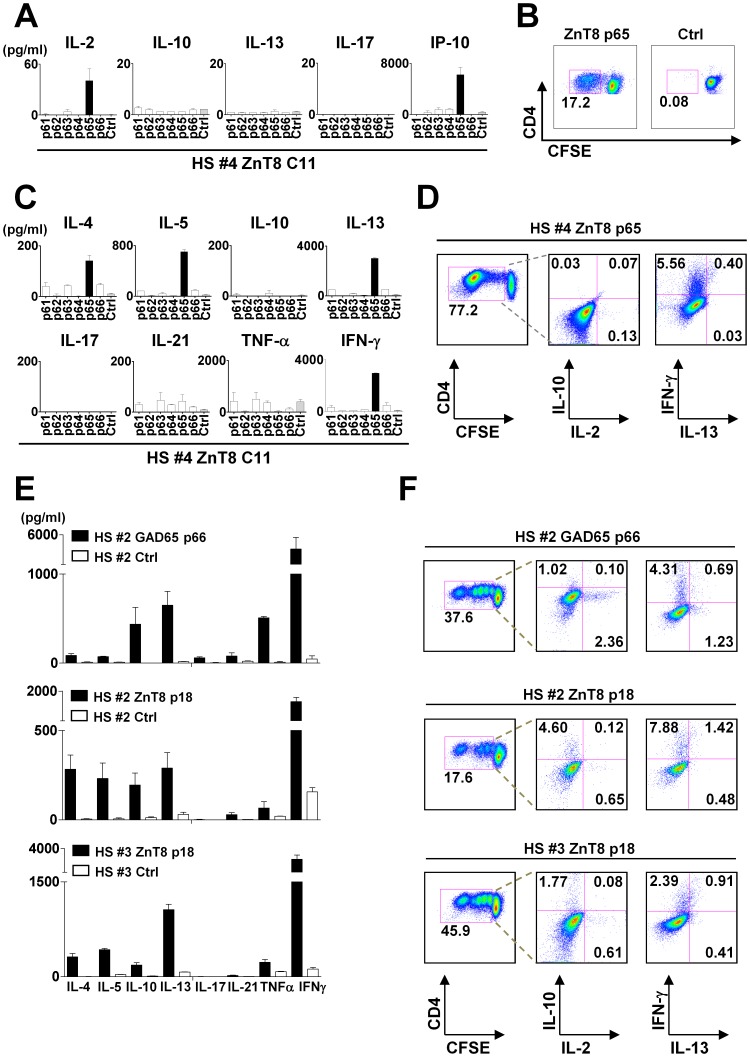
Cytokine secretion profiling of specific CD4^+^ T cells by the Cytokine-driven assay. (**A**) Cytokine secretion by ZnT8 p65-specific T cells in HS#4. Results from the Direct assay. (**B**) ZnT8 p65-specific CD4^+^ T cell proliferation in HS#4. (**C–D**) The Cytokine-driven assay. CFSE-labeled PBMCs from HS#4 were cultured with single peptides from ZnT8 C#11 for 7 days in the presence of IL-2 (day 2–7). (**C**) Cytokine secretion during 24 h re-stimulation with peptides. (**D**) ICS assay after 6 h re-stimulation in the presence of brefeldin A. Gated to CFSE-diluted CD4^+^ T cell populations. (**E-F**) Cytokine secretion profiling of islet-antigen-specific CD4^+^ T cells in healthy adults by the Cytokine-driven assay. (**E**) Cytokine secretion during 24 h re-stimulation with peptides. (**F**) ICS assay after 6 h re-stimulation in the presence of brefeldin A.

We first examined whether the Cytokine-driven assay identifies the same epitopes as the Direct assay. In donor HS#4, the Direct assay identified ZnT8 p#65 from C#11 as the epitope for CD4^+^ T cells by induction of IL-2 and IP-10 secretion (Fig. 4A) and by CD4^+^ T cell proliferation (Fig. 4B). Consistently, the Cytokine-driven assay showed that only p#65 among the six peptides from C#11 induced IFN-γ secretion. p#65-responding T cells were also found to secrete type 2 cytokines, IL-4, IL-5, and IL-13 (Fig. 4C). ICS assay with the expanded p#65-specific T cells confirmed the expression of IFN-γ by CD4^+^ T cells upon restimulation with the peptide (Fig. 4D). IL-13 was also expressed by a minor fraction of IFN-γ-expressing cells. Thus, the results of the Direct assay and the Cytokine-driven assay were consistent, as both assays identified ZnT8 p#65-specific Th1 cells. The Cytokine-driven assay allowed for a more detailed cytokine secretion profiling, as the assay demonstrated a fraction of ZnT8 p#65-specific CD4^+^ T cells that were capable of secreting type 2 cytokines.

We next performed the Cytokine-driven assay on all the GAD65 and ZnT8-specific CD4^+^ T cells that had been identified in the Direct assay. The summary of cytokine secretion profiles is shown in Table 3. While all of the epitopes were identified by the induction of IP-10 in the Direct assay, indicative of IFN-γ secretion, expanded CD4^+^ T cells were found to secrete variable types of cytokines in addition to IFN-γ (Fig. 4E and 4F). For example, GAD65 p#66-specific CD4^+^ T cells in donor HS#2 were found to secrete IL-10, IL-13, and TNF-α (Fig. 4E, top). The ICS assay showed that GAD65 p#66-specific CD4^+^ T cells were composed of IFN-γ^+^IL-13^−^ Th1 cells, IFN-γ^−^IL-13^+^ Th2 cells, and IL-10-producing cells (Fig. 4F).

**Table 3:** Summary of cytokine secretion profiles in the Cytokine-driven assay.

Subject	Antigen	Epitope	Secreted Cytokine								
			IL-4	IL-5	IL-10	IL-13	IL-17	IL-21	TNF-α	IFN-γ	
HS#2	GAD65	p66	–	–	^+^	^+^	–	–	^+^	^+^	
	ZnT8	p2	^+^	^+^	^+^	^+^	–	–	^+^	^+^	
		p18	^+^	^+^	^+^	^+^	–	–	–	^+^	
		p33	^+^	^+^	–	^+^	–	–	–	^+^	
		p93	^+^	–	–	^+^	–	–	^+^	^+^	
HS#3	GAD65	p73	–	^+^	^+^	^+^	–	–	–	^+^	
	ZnT8	p2	^+^	^+^	–	^+^	–	–	–	^+^	
		p18	^+^	^+^	^+^	^+^	–	–	–	^+^	
HS#4	GAD65	p73	–	–	–	^+^	–	–	–	^+^	
	ZnT8	p18	–	–	–	^+^	–	–	–	^+^	
		p65	^+^	^+^	–	^+^	–	–	–	^+^	
		p68	–	^+^	–	^+^	–	–	–	^+^	
HS#7	ZnT8	p65	–	^+^	–	^+^	–	–	–	^+^	
		p68	–	–	–	^+^	–	–	–	^+^	
		p87	–	–	–	^+^	–	–	–	^+^	

HS, healthy subject; p, peptide.

All the fifteen GAD65 and ZnT8 peptides that induced IFN-γ secretion by specific CD4^+^ T cells were also able to induce IL-13 secretion (Table 2). Five peptides in 2 adult samples induced IL-10 secretion, while 10 peptides in 4 adult samples induced IL-4 and/or IL-5. Thus, GAD65- and ZnT8-specific CD4^+^ T cells in healthy adults are capable of secreting multiple types of cytokines, including type 1 and type 2 cytokines as well as IL-10.

### Peptide-reactive T cells Detectable by the Direct Assay Display Higher Avidity than Those Detectable by the Cytokine-driven Assay

**Figure 5:**
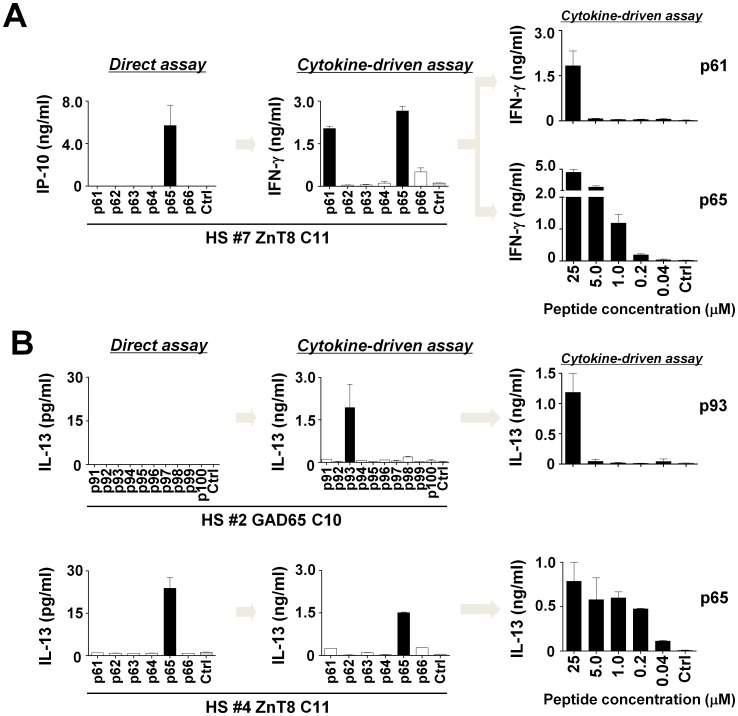
Islet-antigen-specific T cells identified by the Direct assay display higher avidities. (**A**) Left: The results of the Direct assay and the Cytokine-driven assay with ZnT8 single peptides from C#11 in HS#7. Right: PBMCs were cultured with titrated concentrations of the indicated peptides (0.04–25 µM) in the presence of IL-2 (days 2–7), and re-stimulated cells at a constant concentration (25 µM). IFN-γ secretion after re-stimulation. (**B**) Left: The results of the Direct assay and the Cytokine-driven assay with GAD65 single peptides from C#10 in HS#2 (top) and with ZnT8 single peptides from C#11. Right: PBMCs were cultured with titrated concentrations of the indicated peptides in the presence of IL-2 (days 2–7), and re-stimulated cells with 25 µM peptide. IL-13 secretion after re-stimulation.

Using the Direct and Cytokine-driven assays with 25 µM peptide, we found multiple peptides that induced cytokine secretion only in the Cytokine-driven assay. For example, in donor HS#7, while ZnT8 p#61 and p#65 peptides elicited equivalent levels of IFN-γ secretion in the Cytokine-driven assay, ZnT8 p#61 did not induce IP-10 secretion in the Direct assay (Fig. 5A, left and middle). It is possible that ZnT8 p#61-specific CD4^+^ T cells are present in blood at a very low frequency and become detectable only after expansion. Another possibility is that ZnT8 p#61-specific CD4^+^ T cells display an avidity lower than ZnT8 p#65-specific CD4^+^ T cells and secrete little IFN-γ during the initial stimulation, but produce larger amounts of IFN-γ after expansion with IL-2. In this case, low-avidity CD4^+^ T cells would require higher concentrations of peptides than high-avidity CD4^+^ T cells to initiate cell proliferation. To test this hypothesis, we titrated the peptides (from 0.04 to 25 µM) at the initial cultures, and re-stimulated cells at a constant concentration (25 µM). In donor HS#7, while p#61 induced specific IFN-γ secretion only at 25 µM, p#65 induced specific IFN-γ secretion at ∼1 µM (Fig. 5A, right). This suggests that p#65-specific CD4^+^ T cells display higher avidity than p#61-specific CD4^+^ T cells. Similarly, in donor HS#2, while GAD65 p#93 failed to induce cytokine secretion in the Direct assay, but induced IL-13 secretion in the Cytokine-driven assay. A titration experiment showed that 25 µM GAD65 p#93 was required to expand the specific CD4^+^ T cells (Fig. 5B, top). In contrast, only a minute amount of ZnT8 p#65 (<0.2 µM) in HS#4 was sufficient to elicit specific IL-13 secretion (Fig. 5B, bottom).

These results show that the Direct assay identifies T cells with relatively high avidities, while the Cytokine-driven assay can identify antigen-specific T cells including with lower avidities.

### ZnT8-specific CD4^+^ T cells Display Distinct Cytokine Expression Patterns between T1D Patients and Healthy Adults

Previous studies reported that islet antigen, GAD65-specific CD4^+^ T cells with high antigen avidity are prevalent in peripheral blood of T1D patients [8], [33]. Here, we compared the ZnT8-specific T cell repertoires between T1D patients and non-diabetic healthy adults, by using our integrated assay. Herein we used a lower peptide concentration (2.5 µM) than in the experiments described above, in order to detect only T cells with high avidity. Age and gender-matched 15 recent-onset T1D patients (days from diagnosis: 285.9±247.9) and 15 control subjects were accrued in the study (Table 4 and Table S2). HLA distribution based on the frequency of HLA-DRB1*0301, DRB1*0401, and other DR3/DR4 were not statistically different between two groups (*p* = 0.60, Pearson’s χ^2^-test).

**Table 4:** Clinical characteristics of T1D patients and HLA-matched controls.

	T1D	Ctrl
*n.*	15	15
Age (years)	37.4±11.8	37.2±12.4
Gender (M/F)	8/7	8/7
Disease duration (days)	285.9±247.9	NA
HLA-DRB1*0301+ (DQB1*0602-)	4	4
HLA-DRB1*0401+ (DQB1*0602-)	5	2
Other HLA-DR4+	2	3
HLA-DR3-DR4- or DR3+DQB1*0602+	4	6

T1D, Type 1 diabetes patients; Ctrl, HLA-matched controls; n., number;

M, male; F, female; NA, not applicable.

**Figure 6:**
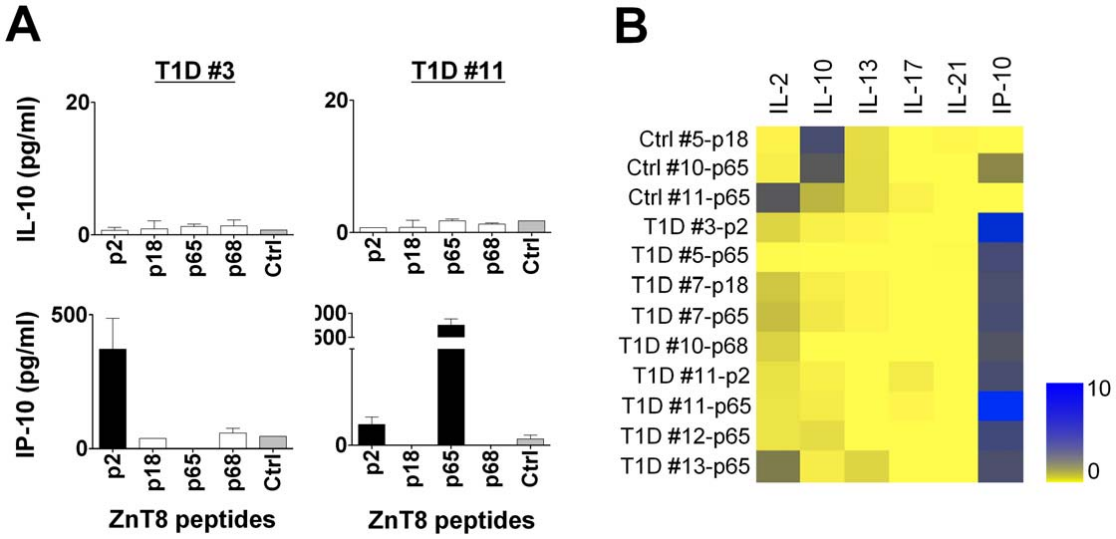
Cytokine secretions from ZnT8-specific T cells in T1D patients and healthy adults in the Direct assay. PBMCs from 15 T1D patients and 15 age/gender/HLA-matched controls were stimulated with 2.5 µM of ZnT8 single peptides (p#2, p#18, p#65, and p#68) in duplicates for 48 hours and six cytokines secreted during the stimulation were measured. (**A**) Representative results of ZnT8-specific IL-10 and IP-10 secretion with T1D patient samples. (**B**) Summary of cytokine secretion patterns. The cultures that scored positive in any cytokines were selected. Data was transformed into a heat-map format indicating the fold increase from the background.

PBMCs were cultured with 2.5 µM of the identified four ZnT8 peptides (p2, p18, p65, and p68), and cytokines secreted during 48 hours culture were measured (Direct assay). With 2.5 µM peptides, none of the PBMC samples from healthy adults produced IP-10 (Fig. 6A, 6B **.** The data summary is shown in Table 5), in contrast to the observations with 25 µM peptides (Fig. 1). Seven out of 15 T1D PBMC samples produced IP-10 in response to at least one of the four ZnT8 peptides (Fig. 6A, 6B). Two out of 15 control PBMCs were found to produce IL-10, while T1D PBMCs produced little IL-10 (Fig. 6A, 6B)**.**

**Table 5:** Number of positive cytokine responses induced by 2.5 µM of ZnT8 peptides.

Peptide #	IL-10 response		IP-10 response	
	T1D	Ctrl	T1D	Ctrl
p2	0	0	2	0
p18	0	1	1	0
p65	0	1	5	0
p68	0	0	1	0

**Figure 7:**
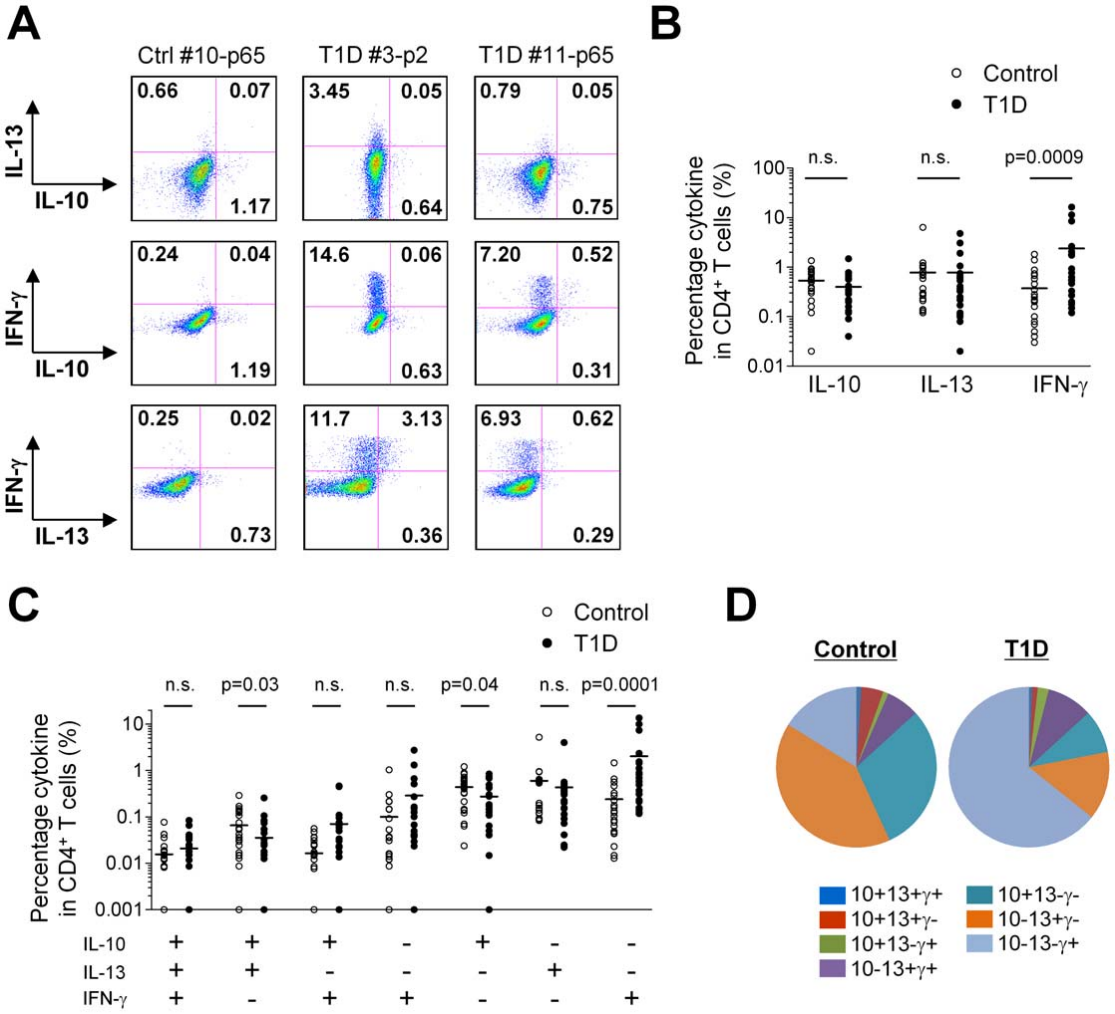
Distinct cytokine expression patterns of ZnT8 specific CD4^+^ T cells between T1D patients and healthy adults. PBMCs from 15 T1D patients and 15 age/gender/HLA-matched controls were cultured with ZnT8 single peptides for 7 days in the presence of IL-2 (day 2–7). ICS assay was performed after 6 h re-stimulation with the same peptides. Gated to CD4^+^ T cells. (**A**) Representative results of cytokine expressions by CD4^+^ T cells specific for ZnT8 single peptides in T1D patients and controls. (**B, C**) Cytokine expression patterns of ZnT8-specific CD4^+^ T cells. 24 from 60 control PBMC cultures (15 subjects×4 peptides) and 22 from 60 T1D PBMC cultures, which contained CD4^+^ T cells expressing any of three cytokines at a frequency of >0.5%, were used for the analysis. Statistical significance between control group and T1D group was tested by Student’s *t*-test. (**D**) Composition of ZnT8-specific CD4^+^ T cells with distinct cytokine-expression pattern in T1D patients and controls.

The cytokine expression pattern of ZnT8-specific CD4^+^ T cells in each group was further analyzed by ICS in the Cytokine-driven assay. We analyzed the expression of IL-10, IL-13, and IFN-γ by CD4^+^ T cells following a short re-stimulation with the peptide. We selected 24 from 60 control PBMC cultures (15 subjects×4 peptides) and 22 from 60 T1D PBMC cultures, which contained CD4^+^ T cells expressing any of the three cytokines at a frequency of >0.5%. A statistical analysis showed that the frequency of IFN-γ-expressing CD4^+^ T cells was higher in T1D PBMC cultures than in control PBMC cultures (Fig. 7A, 7B), a consistent result with the Direct assay (Fig. 6B). Furthermore, we found that control PBMC cultures contain more IL-10^+^IL-13^+^IFN-γ^−^ and IL-10^+^IL-13^−^IFN-γ^−^ cells than T1D PBMC cultures (Fig. 7C). Lastly, we determined the overall cytokine expression pattern of the ZnT8-specific CD4^+^ T cells in T1D patients and controls. The mean frequency of the CD4^+^ T cells with each cytokine secretion pattern was calculated from the selected 24 samples from control and 22 samples T1D PBMC, and used to determine their composition. As shown in Figure 7D, the majority of ZnT8-specific CD4^+^ T cells in T1D displayed the pattern of IL-10^−^IL-13^−^IFN-γ^+^. In contrast, in control subjects, IL-10^+^IL-13^−^IFN-γ^−^ and IL-10^−^IL-13^+^IFN-γ^−^ cells were more prevalent than IL-10^−^IL-13^−^IFN-γ^+^ cells.

These data indicate that the type of ZnT8-specific CD4^+^ T cells is largely distinct between T1D patients and healthy adults. While Th1 cells are dominant in T1D patients, Th2 and IL-10-producing cells are dominant in healthy adults.

## Discussion

In this study, we applied the Direct assay and the Cytokine-driven assay to characterize islet-antigen-specific T cell repertoires in healthy adults and T1D patients.

In the first set of study, where the peptides were used at 25 µM concentration, healthy adults were found to display GAD65- and ZnT8-specific memory Th1 cells at high frequencies (>0.5% of total CD4^+^ T cells). This finding was unexpected, as previous reports demonstrated that islet-antigen-specific T cells in healthy adults were either naïve, anergic, or exhibited regulatory functions [19], [20], [32]. Furthermore, a fraction of ZnT8-specific Th1 cells in healthy adults also co-expressed IL-2. Thus, our study suggests that activation of GAD65 and ZnT8-specific CD4^+^ T cells may occur even in healthy individuals. Importantly, neither PPI-specific CD4^+^ T cells nor islet-antigen-specific CD8^+^ T cells were detected in any tested donors. Failure to detect islet-specific CD8^+^ T cells was not due to the limitation in the assays, as the Cytokine-driven assay was able to detect GAD65-specific CD8^+^ T cells in multiple T1D patients who underwent islet transplantations [34]. Our observation is largely consistent with previous reports concluding that insulin-specific T cells [26], [35] and islet-antigen-specific CD8^+^ T cells [36] are barely found in healthy individuals. Recent studies show that insulin-specific CD8^+^ T cells can kill human pancreatic β cells [37] and insulin or/and IGRP-specific CD8^+^ T cells are present insides the islets of T1D patients [38]. Therefore, the presence of PPI-specific T cells and islet-specific CD8^+^ T cells might be more limited to T1D patients when compared to GAD65- or ZnT8-specific CD4^+^ T cells.

The Cytokine-driven assay showed that GAD65- or ZnT8-specific CD4^+^ T cells in healthy adults were composed of heterogeneous populations that differentially express IFN-γ, IL-10, and type 2 cytokines, including IL-4, IL-5, and IL-13. While IFN-γ secreted by islet-antigen-specific CD4^+^ T cells might be prone to induce islet inflammation [17], [39], IL-10 secreted by T cells might play an inhibitory role [19], [40]. The role of Type 2 cytokine-secreting CD4^+^ T cells in healthy adults is still unclear. A previous study shows that insulin-specific T cell clones established from pancreatic lymph nodes of T1D patients preferentially secreted IL-13, but not IFN-γ [25], suggesting that these cells might play a pathogenic role. Alternatively, it is possible that Type 2 cytokines play an anti-inflammatory role. For example, a systemic administration of recombinant IL-13 prevents the onset of diabetes in NOD mice [41]. Furthermore, generation of IL-5-producing GAD65-specific CD4^+^ T cells was shown to protect NOD mice from diabetes development [42].

In this study, we identified two 15-mer GAD65 peptides (p66 and p73) and eight 15-mer ZnT8 peptides (p2, p8, p18, p33, p65, p68, p87, and p93) as epitopes for CD4^+^ T cells. While we did not test whether CD4^+^ T cells expanded with the identified peptides also react to naturally processed antigens, the two GAD65 peptides and the most ZnT8 peptides shared sequences with CD4^+^ T cell epitopes reported previously [21], [43], [44], [45], [46]. Nonetheless, our approach also has limitations. While 25 µM ZnT8 peptides induced strong IP-10 production in the PBMC cultures from healthy adults, 2.5 µM peptides did not. This discrepancy might be due to the activation of CD4^+^ T cells by large amounts of peptides, with relatively low avidity or potentially cross-reactive. Furthermore, previous studies show that truncation and extensions of peptide sequences by a single amino acid might change the quality of expanded T cells in terms of cytokine expression profiles [47]. Therefore, suboptimal peptide ligands might alter the outcome. Thus, to determine the type of antigen-specific T cells with this approach, two points should be considered: testing multiple epitope peptides rather than single peptide from a library and using low concentration of peptides.

Finally, we compared the quality of ZnT8-specific T cell responses between healthy adults and T1D patients by using the identified peptides in this study. Herein we used peptides at a 10-fold less concentration than the first set (2.5 µM), to minimize the activation of low-affinity T cells and possible cross-reactivity. We found that ZnT8-specific Th1 cells were more prevalent in T1D patients compared to healthy adults, which is consistent with a recent study [21]. Furthermore, we found that Th2 and IL-10-producing cells were dominant among ZnT8-specific CD4^+^ T cells in healthy controls. This is in line with the previous study demonstrating that IA-2 and proinsulin-specific CD4^+^ T cells in healthy adults secrete IL-10 rather than IFN-γ [19]. Thus, our study further extends this observation, and shows that ZnT8-specific CD4^+^ T cell subsets are distinctly skewed between T1D patients and healthy adults.

In conclusion, our integrated approach is able to determine the breadth, type, and frequency of antigen-specific T cells simultaneously. Application of this approach might facilitate the characterization of antigen-specific T cell repertories in many immune-mediated diseases.

## Methods

### Blood Samples

Peripheral blood samples were obtained from 10 healthy adults (HS #1-#10) in Baylor Research Institute, 15 T1D patients (T1D #1-#15) and 15 age/gender/HLA-matched controls (Ctrl #1-#15) in Benaroya Research Institute, who signed the written informed consent forms. This study was approved by the institutional review board of Baylor Research Institute (project no. 097-027) and Benaroya Research Institute (project no. IRB7109-92). Peripheral blood mononuclear cells (PBMCs) were isolated by density gradient centrifugation, using Ficoll-Paque PLUS (GE-Healthcare Bio-Sciences, Piscataway, NJ) from sodium-heparinized blood within 24 hours after sampling and kept frozen at −80 C until use.

### Overlapping Peptide Libraries

A 15-mer overlapping peptide library was designed to cover the entire amino acid sequence of islet-specific antigens with 11 amino acid (AA) overlaps (BioSynthesis, Lewisville, TX). Three peptide libraries; GAD65 (585 AA, 144 peptides), PPI (110 AA, 25 peptides), and ZnT8 (369 AA, 94 peptides) were used (Table 1). Peptides were dissolved at 10 mM with 50% acetonitrile (Sigma-Aldrich Co., St. Louis, MO) and kept frozen at −80 C.

### Cell Cultures

PBMCs were re-suspended at a concentration of 2.5×10^6^ cells/ml in complete medium (CM); RPMI 1640 medium (GIBCO, Carlsbad, CA) supplemented with 1% L-glutamine (Sigma-Aldrich), 1% penicillin/streptomycin (Sigma-Aldrich), 50 µM 2-β-mercaptoethanol (Sigma-Aldrich), 1% sodium pyruvate (Sigma-Aldrich), 1% non-essential amino acids (Sigma-Aldrich), and 10% heat-inactivated human AB serum (Gemini Bio-Products, Sacramento, CA). The cell viability was examined with 0.4% trypan-blue solution (Sigma-Aldrich) and was always >95%. Five×10^5^ cells per well were cultured in a 96-well deep well plate in the presence of single peptides (0.04–25 µM each peptide) or peptide clusters (pooled 4–11 single peptides/cluster at 10 µM each peptide). An equal amount of peptide diluent was used as a negative control. On day 2 of culture, 80 µl of culture supernatant was harvested for the cytokine secretion assay. To expand the antigen-specific T cells, 100 U/ml recombinant human IL-2 (TECIN [Teceleukin]; Roche, Nutley, NJ) was added to the culture at day 2 of culture. Cells were harvested on day 7 of culture, rested overnight in fresh media, and re-stimulated with the same peptides for 6 hours for an intracytoplasmic cytokine expression assay and 24 hours for the cytokine secretion assay.

### Intracytoplasmic Cytokine Expression Assay

PBMCs were re-suspended with CM at a concentration of 2.5×10^6^ cells/ml, and stimulated with 2.5 or 25 µM of the identified peptides for 6 hours in the presence of CD28/CD49d co-stimulatory antibodies (BD Biosciences, San Jose, CA). Brefeldin A (Golgi Plug™; BD Biosciences) was added for the last 4 hours of culture. After surface staining, cells were fixed, parmeabilized, and then stained for intracytoplasmic cytokines. The used antibodies are listed in Table S3. Stained cells were acquired by a FACSCantoII™ flow cytometer (BD Biosciences) and the data were analyzed with FLOWJO software (Tree Star, Inc., Ashland, OR). CD28/CD49d co-stimulatory antibodies were not used for ICS in the Cytokine-driven assay.

### Cytokine Secretion Assay

Secreted cytokine levels (IL-2, IL-10, IL-13, IL-17A, and IP-10 for the Direct assay; and IL-4, IL-5, IL-10, IL-13, IL-17A, IL-21, TNF-α, and IFN-γ for the Cytokine-driven assay) were measured by a multiplex bead-based cytokine assay (BIO-RAD, Hercules, CA). Data were analyzed using the software GraphPad PRISM. For the visualization of the secreted cytokine levels, the data were transformed into a heat-map format indicating the fold increase from the background.

### CFSE Dilution Assay

CFSE-labeled PBMCs were re-suspended at a concentration of 2.5×10^6^ cells/ml in CM and stimulated with 25 µM single peptides. On day 8 of culture, cells were harvested and then stained with CD8 PE (SK1), CD3 PerCP-Cy5.5 (SK7), and CD4 APC (SK3; eBioscience, San Diego, CA). Proliferating T cell populations were analyzed based on CFSE dilution with FACSCalibur™ or FACSCantoII™ flow cytometers (BD Biosciences).

### Statistical Analysis

Bar graphs represent mean ± SD. Significance of difference between experimental variables was determined using the Student’s *t*-test.

## Supporting Information

Table S1 **Sequence of the identified CD4^+^ T cell epitopes.**
(DOC)

Table S2 **Characteristics of each T1D patients and HLA-matched controls who participated in the study to compare the ZnT8-specific T cell repertoire between the two groups.**
(DOC)

Table S3 **Antibodies for intracytoplasmic cytokine detection assay.**
(DOC)
