# Supplementary material for: ZnT8-Specific CD4+ T Cells Display Distinct Cytokine Expression Profiles between Type 1 Diabetes Patients and Healthy Adults
Source: PLoS One. 2013 Feb 4;8(2):e55595. doi: 10.1371/journal.pone.0055595 (PMC3563599; doi:10.1371/journal.pone.0055595)
Supplement: Table S1 — Sequence of the identified CD4+ T cell epitopes. (DOC) [file pone.0055595.s001.doc]

**Table S1.** Amino acid sequence of the identified peptides

| **Antigen** | **Peptide #** | **Position** | **Amino acid sequence** |
| --- | --- | --- | --- |
| GAD65 | p66 | 261-275 | EVKEKGMAALPRLIA |
|  | p73 | 289-303 | AAALGIGTDSVILIK |
| ZnT8 | p2 | 5-19 | ERTYLVNDKAAKMHA |
|  | p8 | 27-41 | LQQKPVNKDQCPRER |
|  | p18 | 67-80 | YAYAKWKLCSASAI |
|  | p33 | 124-138 | SSKPPSKRLTFGWHR |
|  | p65 | 244-258 | YKIADPICTFIFSIL |
|  | p68 | 254-268 | IFSILVLASTITILK |
|  | p87 | 326-340 | DSQVVRREIAKALSK |
|  | p93 | 352-366 | ESPVDQDPDCLFCED |
