# Supplementary material for: ZnT8-Specific CD4+ T Cells Display Distinct Cytokine Expression Profiles between Type 1 Diabetes Patients and Healthy Adults
Source: PLoS One. 2013 Feb 4;8(2):e55595. doi: 10.1371/journal.pone.0055595 (PMC3563599; doi:10.1371/journal.pone.0055595)
Supplement: Table S2 — Characteristics of each T1D patients and HLA-matched controls who participated in the study to compare the ZnT8-specific T cell repertoire between the two groups. (DOC) [file pone.0055595.s002.doc]

**Table S2.** Characteristics of each T1D patient and HLA-matched controls

| **ID** | **Age (years)** | **Gender** | **Disease duration (days)** | **HLA-DRB1** | **HLA-DQB1** |
| --- | --- | --- | --- | --- | --- |
| Ctrl #1 | 61 | F | NA | *1101*1104/*1501*1502 | *0301/*0602 |
| Ctrl #2 | 58 | F | NA | *1101*1102*1104/*1301*1302*1305 | *0301/*0603 |
| Ctrl #3 | 55 | F | NA | *0101/*0301 | *02/*0501 |
| Ctrl #4 | 51 | M | NA | *1202/*1501*1502 | *0301/*0601 |
| Ctrl #5 | 36 | F | NA | *0101/*0301*0304 | *02/*0501 |
| Ctrl #6 | 35 | M | NA | *0403/*1501*1502 | *0302/*0502 |
| Ctrl #7 | 34 | M | NA | *1301*1302/*1501*1502 | *0603/*06 |
| Ctrl #8 | 32 | M | NA | *0404/*1501*1502 | *0302/*0503 |
| Ctrl #9 | 30 | M | NA | *0405/*1201 | *0301/*0401 |
| Ctrl #10 | 25 | M | NA | *0701/*1101*1104 | *0202*0301 |
| Ctrl #11 | 26 | F | NA | *0101/*1301*1302 | *0301/*06 |
| Ctrl #12 | 28 | M | NA | *0401/*0701 | *0202/*0301 |
| Ctrl #13 | 28 | M | NA | *0301/*1101*1104 | *0201/*0301 |
| Ctrl #14 | 30 | F | NA | *0401/*0701 | *0302*0305/*02 |
| Ctrl #15 | 29 | F | NA | *0301/*0407 | *0201/*0301 |
| T1D #1 | 59 | F | 56 | *0401/*1301*1302 | *0301/*0604 |
| T1D #2 | 56 | F | 251 | *0301*0304/*0301*0304 | *0201/*0201 |
| T1D #3 | 54 | F | 251 | *0301/*1301*1302 | *02/*0604 |
| T1D #4 | 53 | M | 92 | *0401/*0403 | *0302/*0502 |
| T1D #5 | 37 | F | 466 | *0401/*0401 | *0302/*0302*0305 |
| T1D #6 | 36 | M | 42 | *0401*0413/*0404*0408 | *0301/*0302 |
| T1D #7 | 35 | M | 28 | *0101/*0102 | *0501/*0501 |
| T1D #8 | 32 | M | 679 | *0101/*0401 | *0501/*0302 |
| T1D #9 | 31 | M | 729 | *1602/*1302 | *0502/*0604 |
| T1D #10 | 25 | M | 47 | *0101/*0401 | *0501/*0301 |
| T1D #11 | 26 | F | 638 | *0404*0405*0408/*1101*1104 | *03/*03 |
| T1D #12 | 28 | M | 453 | *0103/*0701 | *0202/*0301 |
| T1D #13 | 29 | M | 297 | *0301/*0405 | *02/*0302 |
| T1D #14 | 30 | F | 132 | *0301/*1301*1302 | *0201/*0604 |
| T1D #15 | 30 | F | 127 | *0101*0102/*1601 | *0501/*0502 |

T1D, Type 1 diabetes patients; Ctrl, HLA-matched controls; M, male; F, female; NA, not applicable
