# Supplementary material for: ZnT8-Specific CD4+ T Cells Display Distinct Cytokine Expression Profiles between Type 1 Diabetes Patients and Healthy Adults
Source: PLoS One. 2013 Feb 4;8(2):e55595. doi: 10.1371/journal.pone.0055595 (PMC3563599; doi:10.1371/journal.pone.0055595)
Supplement: Table S3 — Antibodies for intracytoplasmic cytokine detection assay. (DOC) [file pone.0055595.s003.doc]

**Table S3.** Antibodies for intracytoplasmic cytokine detection assay

| **Antibody** | **Clone** | **Supplier** |
| --- | --- | --- |
| CD3 APC-Alexa Fluor 750 | S4.1 | Invitrogen |
| CD4 PE-Cy7 | S3.5 | BD Pharmingen |
| CD8 PerCP | SK1 | BD Pharmingen |
| CD8 Pacific Blue | 3B5 | Invitrogen |
| CD45RO PE-Cy5 | UCHL1 | BD Pharmingen |
| IL-2 FITC | MQ1-17H12 | BD Pharmingen |
| IL-2 APC | MQ1-17H12 | BD Pharmingen |
| IL-10 PE | JES3-19F1 | BD Pharmingen |
| IL-13 PE | JES10-5A2 | BD Pharmingen |
| IL-13 APC | JES10-5A2 | BioLegend |
| IFN-γ PE | 4S.B3 | BD Pharmingen |
| IFN-γ V450 | B27 | BD Pharmingen |
